# The application of myofascial anterolateral thigh flap in reconstruction of oropharyngeal defect: a case report^☆^

**DOI:** 10.1016/j.bjorl.2023.101367

**Published:** 2023-11-20

**Authors:** Shengyou Ge, Xiaochen Yang, Zongxuan He, Wei Shang, Kai Song

**Affiliations:** a The Affiliated Hospital of Qingdao University, Department of Oral & Maxillofacial Surgery, Shandong Province, China; b Qingdao University, School of Stomatology, Shandong Province, China

## Introduction

Owing to the anatomic complexity of the oropharyngeal region, the tumours in this area often invade several tissues, including the base of the tongue, tonsils, oropharyngeal wall, and epiglottis. Tumour reconstruction is required to achieve an anatomical and functional recovery.^1^ Recently, the use of Myofascial Anterolateral Thigh (MALT) flap has been reported in the reconstruction of tongue defects.^2^ However, the role of this flap has not been well studied in the reconstruction of oropharyngeal defects. Herein, we present the 18-month follow-up details a case of oropharyngeal defect reconstruction using a MALT flap, following tumour ablation in a 55-year-old man diagnosed with oropharyngeal Human Papilloma Virus (HPV)-negative Squamous Cell Carcinoma (SCC).

## Case report

**Figure 1:**
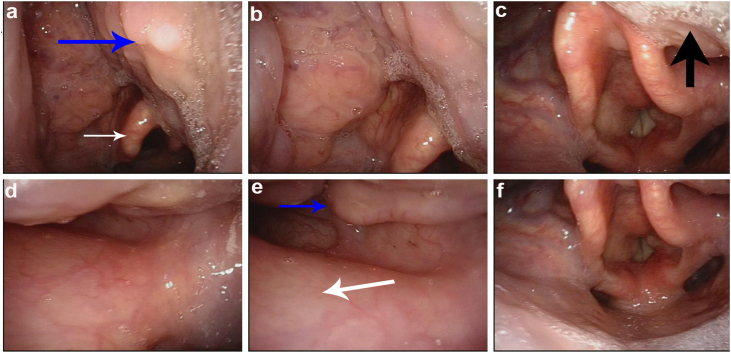
Endoscopic imaging of the location and invasion of tumour. Tumour labeled with blue arrow, the base of tongue with black arrow, epiglottis with white arrow.

A 55-year-old man presented to our outpatient clinic with a six-month history of pharyngalgia and dysphagia. A cauliflower-like neoplasm on the right side of the lateral oropharyngeal wall was detected through transnasal videolaryngoscopic examination (Fig. 1); an immediate biopsy was performed. Histopathology revealed an HPV-negative moderately differentiated SCC.

**Figure 2:**
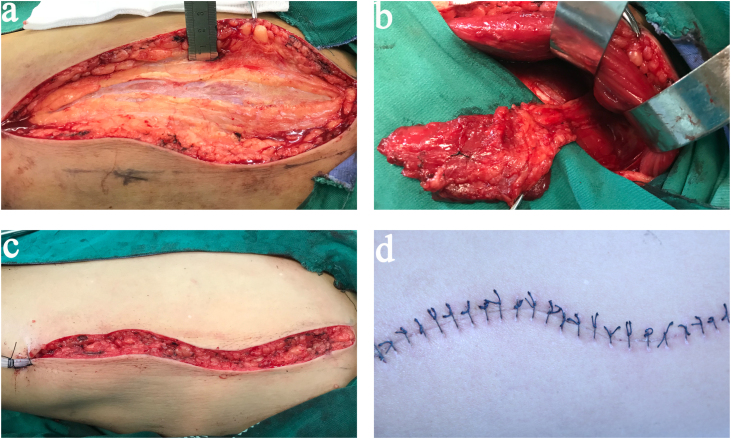
Surgical procedure of the flap preparation. (a) The thickness of excessive bulk of subcutaneous fat and skin fascial was more than 2 cm; (b) Flap raising was finished; (c and d) the photos of donor site.

Ablative surgery was performed with resection of the right tonsil, most of the right lateral oropharyngeal wall, and part of the base of the tongue; ipsilateral modified radical neck dissection was performed in the same session. Thereafter, the oropharyngeal defect was reconstructed using a MALT flap. During the procedure, the vastus lateralis muscle was exposed, elevated, and reshaped as the main segment of the MALT flap (Fig. 2 a and b). The right superior thyroid artery and two branches of the internal jugular vein were used for vascular anastomoses. The flap was sutured to the mucosa adjacent to the defect, with the fascial surface outward; the donor sites were closed primarily without significant tension (Fig. 2 c and d).

**Figure 3:**
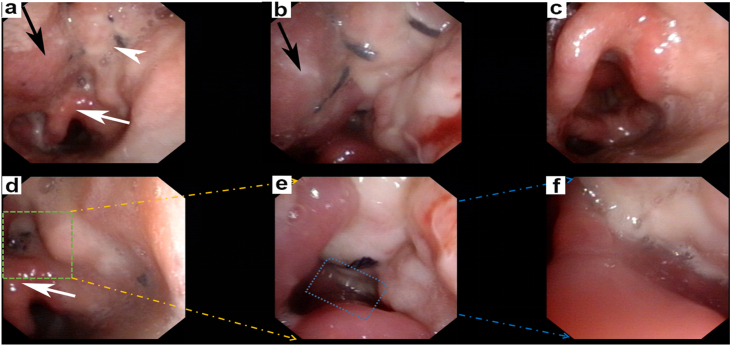
Pharyngorhinoscopy imaging of patient on tenth day after the surgery. (a) The black arrow showed the tongue base; the white arrowhead showed the restoration of soft tissue with myofascial flap; the white arrow showed the epiglottis; (b) The commissure between tongue base (black arrow) and myofascial flap was well; (c) The mobility of epiglottis was well when the patient pronounced the sound of “ei” ; (d, e and f) The commissure between myofascial ALT flap and epiglottis was reconstructed well with smooth and soft texture of surface.

**Figure 4:**
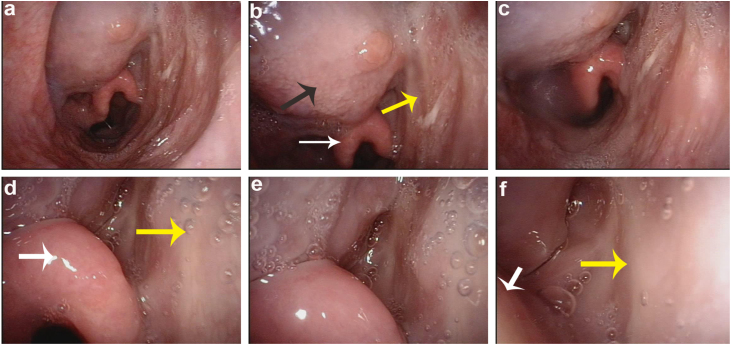
Patient at the six-month follow-up. Flap labelled with yellow arrow, the base of tongue with black arrow, epiglottis with white arrow.

Postoperatively, nasal feeding was used for the patient. On the 3rd day after operation, the patient started with swallowing train by drinking sips of water via mouth, and on the 20th day, the patient accepted fluid diet by mouth and nasal feeding tube was removed. Nasopharyngoscopy was performed at 10 days and 6 months follow-up after surgery, respectively. Ten days after surgery, spontaneous remucosalization was observed on the fascial surface of the MALT flap (Fig. 3). Six months later, adequate mobility of the epiglottis was observed, when the patient pronounced the sound “ei”; the commissure between the lateral wall of the pharynx and epiglottis was reconstructed well (Fig. 4 and Supplemental Digital Content 1). No postoperative complications or tumour recurrence were noted.

## Discussion

Head and neck reconstruction surgery now mainly focused on the application of the fasciocutanesous and musculocutaneous flaps, including the selection and design of flaps, and evaluation of the success of free flaps transplantation. Although different flaps were reported and met high reconstruction demands, the skin paddle of the common used fascio- and myocutanous flaps still present some drawbacks, such as slow healing, unsatisfactory texture, unattractive colour match, excessive bulk of subcutaneous fat and skin, donor morbidity and so on.^2^ These shortcomings may affect the application of free flaps in oropharyngeal reconstruction after tumour ablation.

Following the concept of “replace like with like”, the usefulness of myofascial flaps have been reported and gained more and more attention. The use of the pectoralis myofascial flap in the reconstruction of defects of the head and neck was proposed 20 years ago. Gras et al. considered this flap to be useful for soft tissue coverage after oropharyngeal and pharyngolaryngeal tumour ablation.^3^ The idea that application of temporalis myofascial flap for successfully reconstructed palatal defect was the foundation of studying myofascial flap epithelialization or remucosalisation and provided a foundation for future application of the vascularized myofascial flap in head and neck reconstruction.^4^

In recent years, the MALT flap has been reported as a novel approach to reconstruction of the tongue and floor of the mouth.^5^ The MALT flap can overcome few limitations of the common ALT flap, such as thickness and enlargement in patients with a high body neoplasm index, variation of cutaneous perforators, and hair bearing in male patients.^5^ However, no studies have confirmed whether the MALT flap is suitable for the reconstruction of oropharyngeal defects, particularly when tumours invade the base of the tongue and the oropharyngeal wall. This report describes the case of a 55-year-old man who was diagnosed with oropharyngeal HPV-negative SCC and achieved a functional recovery after a MALT flap reconstruction.

In this case, vascularised MALT flap could be reshaped easily and provided a guided condition to allow spontaneous remucosalization in the oropharyngeal defects. Six months after the surgery, the gross appearance of the reconstructed oropharynx was identical to that of the residual mucosa; the epiglottic valley and piriform fossa were revealed clearly as well. Meanwhile, no dysphonia or dysphagia was noted during the follow-up. We hypothesise that this reliable method may contribute to favourable deglutition and phonation, owing to the acceptable contraction and smooth and wet flap surface.

The MALT flap appears to be a novel and reliable reconstruction choice for a subgroup of patients after oropharyngeal cancer resection. Further in-depth studies are recommended to evaluate the efficacy and safety of this flap.

## Conclusion

Oropharynx reconstruction following cancer resection remains one of the most challenging problems owing to the anatomic complexity in this region. Although the ALT flap has become a popular flap for oral and oropharyngeal reconstruction, several disadvantages still exist in clinical practice. The MALT flaps are suitable for oropharyngeal tumour reconstruction, particularly when tumours invade the base of the tongue and the oropharyngeal wall. By our preliminary experience, this report may provide further guidance for surgeons to restore the oropharyngeal defects in a reliable and versatile approach.

## Funding

This work was supported by the National Natural Science Foundation of China (grant numbers 81502340).

## Patient consent

Written patient consent was obtained to publish the clinical photographs.

## Conflicts of interest

The authors declare no conflicts of interest.
