# Supplementary material for: The application of myofascial anterolateral thigh flap in reconstruction of oropharyngeal defect: a case report
Source: Braz J Otorhinolaryngol. 2023 Nov 20;90(2):101367. doi: 10.1016/j.bjorl.2023.101367 (PMC10698541; doi:10.1016/j.bjorl.2023.101367)
Supplement: Supplementary file 1 [file mmc1.doc]

**BJORL-D-23-00175_Supplementary Material**

**Supplemental Digital Content 1 Pharyngorhinoscopy video of the patient during the perioperative and follow-up period.** Pharyngorhinoscopy examination revealed adequate information, such as the location of the oropharyngeal tumour, a gradual spontaneous and centripetal remucosalization was observed on the fascial surface of the MALT flap 10 days after surgery, and favourable functional results at the six-month follow up visit.
